# Randomized controlled trials: still the backbone of vascular surgery?

**DOI:** 10.1007/s00772-015-0100-z

**Published:** 2015-12-10

**Authors:** A.R. Naylor

**Affiliations:** The Department of Vascular Surgery, Vascular Research Group, Division of Cardiovascular Sciences, Clinical Sciences Building, Leicester Royal Infirmary, LE27LX Leicester, United Kingdom

**Keywords:** Randomized trials, Observational studies, Vascular surgery, Treatment outcome, Meta-analysis, Randomisierte Studien, Beobachtungsstudien, Gefäßchirurgie, Behandlungsergebnis, Metaanalyse

## Abstract

Prior to the introduction of evidence-based medicine, decision-making was largely based upon ‘intuitive reasoning’, whereby senior clinicians dictated practice based upon personal dogma, personal experience and (often) biased observational studies. This era began to end (in vascular surgery) following completion of the landmark randomized trials in carotid disease, which recruited patients throughout the 1980s. Despite scepticism amongst some surgeons of the time these particular randomized trials have stood the test of time and remain the cornerstone of virtually every guideline of practice to this day. The carotid randomized trials became a beacon for using ‘evidence’ rather than ‘intuitive reasoning’ and randomized trials have now been used to determine optimal practice in a plethora of carotid surgery and stenting trials, lower limb revascularization and numerous aortic aneurysm based studies.

The literature abounds with situations where practice (previously based on observational study data) was changed overnight following publication of a well-designed randomized trial. However, while observational studies are prone to selection bias, randomized trials bring their own unique limitations including problems with external validity, they take too long to complete, they are very expensive, they are notorious for problems with recruitment and they can frequently become obsolete. This has led to a (not unreasonable) call for more observational studies to be used in the development of practice guidelines. Unfortunately, the principle guideline bodies around the world, e.g. National Institute for Health and Care Excellence (NICE) and the American Heart Association (AHA), prioritize randomized trial evidence above all else. Until that changes, guideline makers will find it very difficult to deviate from using historical randomized trial evidence, even when high quality observational data suggest that ‘real world’ practice bears little comparison to that reported in the randomized trials. Nowhere is that more evident than in developing contemporary guidelines for the management of asymptomatic carotid disease.

## Abstract (de)

Vor der Einführung der evidenzbasierten Medizin basierten Entscheidungen weitgehend auf Intuition, wobei die ärztlichen Vorgesetzten die praktische Vorgehensweise auf der Grundlage ihrer persönlichen Dogmen und Erfahrung sowie (oftmals) verzerrter Beobachtungsstudien diktierten. Der Anfang vom Ende dieser Ära wurde eingeleitet durch den Abschluss der wegweisenden randomisierten Studien zu Karotiserkrankungen, für welche Patienten in den 1980er-Jahren rekrutiert wurden. Trotz der Skepsis mancher Chirurgen damals haben sich gerade diese randomisierten Studien bewährt und bilden bis heute immer noch den Grundpfeiler praktisch jeder Praxis-Leitlinie. Die randomisierten Studien zu Karotiserkrankungen wurden ein leuchtendes Beispiel für die Verwendung von „Evidenz“ statt „Intuition“, und seitdem werden randomisierte Studien zur Ermittlung des optimalen Vorgehens in einer Vielzahl von Studien zur Karotischirurgie, zur Stenteinlage, zur Revaskularisierung der unteren Extremität und in zahlreichen Untersuchungen zu Aortenaneurysmen eingesetzt.

Es finden sich in der Literatur viele Situationen, in denen die Praxis (die vorher auf Daten von Beobachtungsstudien beruhte) nach Publikation einer gut konzipierten randomisierten Studie über Nacht geändert wurde. Während Beobachtungsstudien für eine Verzerrung durch Selektion anfällig sind, weisen randomisierte Studien jedoch eigene spezielle Limitationen auf, einschließlich Problemen mit externer Validität, langer Dauer bis zum Abschluss, hoher Kosten, der bekannten Schwierigkeiten bei der Rekrutierung und dass sie dann häufig überholt sind. Das führte zu dem (nicht unverständlichen) Ruf danach, dass mehr Beobachtungsstudien für die Entwicklung von Praxis-Leitlinien eingesetzt werden sollten. Leider geben überall auf der Welt die wesentlichen Leitliniengremien, z. B. das National Institute for Health and Care Excellence (NICE) und die American Heart Association (AHA) randomisierten Studien den Vorzug vor allem anderen. Bis sich das ändert, werden die Entwickler von Leitlinien es sehr schwer haben, von der Verwendung von Evidenz aus historischen randomisierten Studien abzuweichen, selbst wenn Beobachtungsdaten hoher Qualität Hinweise darauf geben, dass die tatsächliche Praxis mit derjenigen, die in randomisierten Studien beschrieben wird, nur wenig gemein hat. Nirgendwo zeigt sich das deutlicher als bei der Entwicklung zeitgemäßer Leitlinien zur Behandlung asymptomatischer Karotiserkrankungen.

## Introduction

It is important to remember why this debate is taking place. Prior to 1985 ‘intuitive reasoning’ dominated decision-making with senior surgeons determining investigative and therapeutic strategies with little regard for evidence (or risk). A ‘tipping point’ occurred in the mid-1980s regarding the optimal management of patients with symptomatic carotid disease. As far as neurologists (of the time) were concerned surgeons had no accountability, undertook no meaningful audit, published implausibly good results, were largely unwilling to participate in clinical trials and were responsible for huge increases in carotid endarterectomy (CEA) numbers, regardless of appropriateness. The gulf between surgeons and neurologists was highlighted in a Rand Corporation report, which showed that (following independent review), the indication for 65 % of Medicare patients undergoing CEA in 1981 was deemed ‘uncertain’ or ‘inappropriate’. To make matters worse, these patients incurred a 9.5 % risk of death or stroke following surgery [1].

Charles Warlow, an arch-opponent of ‘intuitive reasoning’ and soon to become principle investigator of the European Carotid Surgery Trial (ECST [2]), opined that “surgeons concluded that CEA was of value because they: (i) ignored minor peri-op strokes, (ii) used the worst possible natural history studies for comparison and (iii) did not include strokes following angiography” when justifying a role for CEA. It would be fair to say that many surgeons (of that era) disagreed with his opinion and were highly suspicious of neurologists’ motives and were (to a large part) hostile to using randomized controlled trials (RCTs) to determine ‘best practice’. In short, surgeons believed that the neurologists were simply out to stop them from operating. Paradoxically, however, the very fact that a sufficiently large number of enlightened surgeons and neurologists on both sides of the Atlantic, thereafter participated in the two landmark RCTs in symptomatic patients was actually to become the savior of the operation. Without the ECST and the North American Symptomatic Carotid Endarterectomy Trial (NASCET [3]), CEA would never have risen to the universally accepted position that it currently holds. Not surprisingly, vascular surgeons quickly embraced the move towards evidence-based medicine (EBM), having seen how it had finally rid them of those ‘troublesome neurologists’. Since then, RCTs have been used in the evaluation of a large number of cardiovascular treatments, to the extent that many contemporary surgeons cannot remember (would not know) just how bad the pre-RCT days really were. The current problem, however, is that RCTs (themselves) are becoming a target for increasing criticism, as a growing body of clinicians argue for a return to using observational studies and registries to determine optimal practice.

## What is evidence-based medicine?

The ultimate goal of EBM is the delivery of optimal clinical care to patients, based upon the following tenets: (i) the accumulation of evidence through research and scientific review, (ii) the preparation and dissemination of evidence-based clinical guidelines, (iii) active implementation of evidence-based clinical practice and (iv) subsequent audits to see how closely ‘real world’ practice mirrors that of the supporting RCT evidence [4]. In addition, in a world of increasing cost constraints, EBM is seen as a means of controlling expenditure. Central to EBM is how the scientific evidence is gathered and this is where much of the debate now lies. Put simply, should EBM rely primarily on RCT data (as it has done since the 1980s) or might there now be an increasing role for observational studies or other innovative research strategies?

### Observational studies

It is reasonable to concede that the observational studies of the pre-ECST/NASCET era bear little methodological resemblance to those of the current era. Observational studies do not randomize patients but they do ‘observe’ or document differences in outcomes after management decisions have been made [5]. Observational studies are often used to evaluate the effectiveness of an intervention in the ‘real world’, especially in populations that are under-represented in RCTs (e.g. the elderly, ethnic minorities, women, low socioeconomic status and multiple comorbidities) [6]. They are also useful for formulating hypotheses that can then be tested in future RCTs, for determining sample sizes and for identifying patient subgroups which might benefit from alternative treatment strategies [5].

Observational studies are, however, limited by the fact that ‘experimental groups’ and ‘control groups’ are not matched for patient characteristics (i.e. there is considerable potential for selection bias). Unless carefully designed, it can be difficult to separate effects attributed to the new treatment under investigation from other confounding factors, which may or may not have been equally/unequally distributed between the experimental and control groups. In addition, whilst administrative datasets may provide large numbers of patients for study, they often do not contain sufficient detail regarding comorbidities etc. to allow risk stratification and meaningful statistical evaluation. However, notwithstanding these methodological limitations, RCTs cannot always be undertaken (evidence for developing clinical guidelines in rare clinical conditions has to come from observational studies) and it should be recognized that observational studies have been responsible for discovering causal associations between smoking and lung cancer, asbestos with mesothelioma and thalidomide with birth defects.

### Randomized controlled trials

The fundamental advantage of the RCT is its excellent ‘internal validity’. This means that because all trial participants are randomized, selection bias is greatly minimized in the hope that the only difference between the treatment arms is their exposure to the management strategy under investigation [6]. Reducing or abolishing selection bias then enables more statistically robust analyses; however, whilst RCTs have been integral in determining optimal practice in the management of vascular patients with symptomatic (asymptomatic) carotid disease and for endovascular aneurysm repair (EVAR), there is a growing concern that RCTs have failed to guide more contemporary clinical practices. For example, 13 RCTs have now compared CEA with carotid artery stenting (CAS) with little evidence of consensus; however, many of the flaws or failings in recent RCTs were mostly self-inflicted and should have been anticipated and/or corrected during the planning phase. In retrospect, ECST and NASCET ‘worked’ because they addressed a simple question (comparing two long-established treatment strategies), a sufficiently large number of surgeons and neurologists recognized the need to commit to such a trial and both trials were sufficiently powered to answer the question posed and also enable clinically meaningful subgroup analyses [7, 8].

Unfortunately, the ‘knee-jerk’ response to any contemporary clinical problem is to immediately demand that a randomized trial be performed (usually by a clinical specialty under threat) and this has led to a proliferation of underpowered, underfunded and ill-thought-out RCTs. Accordingly, it has become much too easy to simply ‘shoot the RCT messenger’ rather than address the underlying methodological problems. Put simply, if an RCT (or indeed any observational study) is designed badly, do not be surprised if it becomes ‘part of the problem’.

## Observational studies versus RCTs?

### Meta-analyses of observational study versus RCT data

As a consequence of the problems associated with observational studies preceding the ECST/NASCET in 1991, it is still intuitively believed that observational studies are methodologically inferior to RCTs; however, large scale ‘meta-analyses of meta-analyses’ have observed that this assumption may be ill-founded. In a 2014 Cochrane review, which analyzed the results of healthcare outcomes obtained using observational study designs compared with those obtained from RCTs, Anglemyer et al. identified 14 reviews with data from 1583 meta-analyses, covering 228 different medical conditions with the mean number of studies included in each meta-analysis being 178 [9]. The Cochrane review concluded that “there was little evidence for significant effect estimate differences between observational studies and RCTs”. In addition, they observed that “factors other than study design per se needed to be considered when exploring reasons for a lack of agreement between results of RCTs and observational studies, especially levels of heterogeneity in meta-analyses of RCTs or observational studies”.

### When have observational findings been reversed following RCTs?

Notwithstanding the findings of the 2014 Cochrane review (and before everyone assumes that observational studies should immediately replace all RCTs), it is important to remember that history is littered with examples of where observational studies suggested an important clinical benefit, only for it to be proved false following publication of an RCT. An extensive list of these has been highlighted by Rothwell [10] including high-dose oxygen therapy in neonates, antiarrhythmic drugs after myocardial infarction, fluoride treatment for osteoporosis, bed rest in twin pregnancies, hormone replacement therapy in preventing thrombotic cardiovascular disease, extracranial to intracranial arterial bypass surgery for  stroke prevention in patients with a carotid occlusion, high-dose aspirin therapy prior to CEA and digoxin after myocardial infarction. To these can also be added bone marrow transplantation for the treatment of breast cancer, in the SAMMPRIS trial (which showed no evidence of benefit for intracranial stenting over medical therapy alone in patients with recent neurological symptoms and intracranial disease) [11] and the GALA trial [12]. The GALA trial is an important example of this phenomenon. A meta-analysis of 41 observational studies (25,000 CEAs) reported that CEA under locoregional anesthesia was associated with a 40 % relative risk reduction in perioperative stroke/death compared to CEA patients using general anesthesia, as well as significant reductions in myocardial infarction (MI) and pulmonary complications [13]. However, a 2013 Cochrane review subsequently analyzed data from GALA and 13 other RCTs (4596 patients) and found no evidence that the type of anesthesia influenced perioperative outcomes [14].

### Why do discrepancies occur?

According to Hannan [5] most discrepancies between observational studies and RCTs can be attributed to one or more problems relating to: (i) *selection bias* (less of a problem with RCTs because of the randomization process) which is a major problem for observational studies. The latter can, however, be reduced by careful risk adjustment through regression or propensity scoring: (ii) *problems with generalizability*. All RCTs have very strict inclusion and exclusion criteria (which prevents selection bias and thus increases internal validity) but the findings may not be applicable to a much broader population (i.e. RCTs can lack external validity). This problem will be further compounded if RCTs were undertaken in large, academic hospitals by highly experienced clinicians, if the majority of procedures are actually done by less experienced clinicians in smaller institutions. Paradoxically, observational studies have greater external validity, as they tend to be more representative of ‘real world’ practice: (iii) *inadequate statistical power*. This is a crucial problem. By their very nature, RCTs are expensive, they take a long time to recruit and most usually randomize only a small minority of screened patients (0.5 % of patients undergoing CEA in North America in 1989 were randomized within NASCET [15]).

### Are RCTs always the perfect solution?

Absolutely not: RCTs work best when they are designed to answer an important (preferably simple) clinical question that has a clearly defined (and relevant) clinical endpoint, which is adequately funded and where there is a realistic possibility of recruiting an adequate number of participants in order for it to be adequately powered and externally valid. The problems (and there are many) arise when one or more of these caveats are compromised.

A number of examples of ‘failed’ or ‘flawed’ RCTs in vascular/endovascular practice have been highlighted by Frank Veith [16]. Some of his criticisms are valid, some are open to interpretation, while some represent a triumph of ‘spin over reality’. Examples include problems relating to: (i) *one treatment under evaluation is still evolving*. This has been a particular problem with the CEA vs CAS trials and it is conceded that this is a difficult issue to resolve, because most RCTs take a long time to meet ethical scrutiny and completion: (ii) *there is an improvement in outcomes in the ‘control’ group*. For example, the recent reduction in annual stroke rates on medical therapy in asymptomatic patients with carotid disease probably renders the landmark asymptomatic randomized trials that were undertaken in the 1990s obsolete [17], raising the inevitable question as to whether they should be repeated: *(iii) comparing highly experienced practitioners in one treatment limb with less experienced practitioners in the other*. This has been a particular criticism of the European RCTs comparing CEA with CAS but, on closer scrutiny, this is a classic example of ‘spin over substance’. In the French EVA-3S trial the highest complication rates were observed in the most experienced CAS practitioners [18], while the most experienced centers in the ICSS study also incurred the highest procedural death/stroke rates [19]: *(iv) imperfect randomization strategies*. This is largely a historical problem but is especially relevant when a clinician can predict who will be randomized to each strategy (e.g. randomizing by odd/even date or alternating weeks). This accusation has, however, also been leveled at the IMPROVE trial [20] which randomized patients with a suspected ruptured abdominal aortic aneurysm (rAAA) *before* they underwent a confirmatory computed tomography (CT). This represents a pragmatic trial design strategy but the conclusions of the IMPROVE trial (i.e. no difference in early mortality rates between EVAR and open repair) were the same as those found in several other RCTs which only randomized patients *after* a diagnosis of rAAA was made and where the patients were deemed suitable for both EVAR and open repair [21, 22]. Meta-analyses of 1-year data from the three main European RCTs, however, now show a clinical and cost-effectiveness benefit favoring EVAR [23] and it will be interesting to see whether the volume of criticism by EVAR advocates now diminishes: *(v) recruiting an inappropriate population*. The classic example of this is the SAPPHIRE trial, which randomized (predominantly asymptomatic) patients who were deemed at ‘*high risk for CEA*’ to undergo CAS or CEA. Perhaps not surprisingly, SAPPHIRE concluded that CAS was ‘not inferior’ to CEA [24]; however, the main conclusion should have been that with the high complication rates observed, both treatment groups would have been safer with medical therapy [25] and (vi) *inappropriate endpoints*. This largely refers to the inclusion of non-standard composite endpoints (e.g. death/stroke/MI and/or target lesion revascularization) and surrogate markers. The inclusion of composite endpoints and surrogate markers are accepted to be ‘softer endpoints’ and are included in order to reduce the number of patients to be recruited (i.e. making the trial easier and cheaper to complete). The inclusion of ‘MI’ within the composite endpoint of perioperative death/stroke/MI in the CREST trial (comparing CEA vs. CAS) has been heavily criticized (usually by surgeons), because a subgroup analysis from CREST subsequently concluded that CAS was associated with a twofold reduction in perioperative MI, which was then associated with poorer long-term mortality rates [26]. In fact, while CEA was associated with a higher rate of perioperative MI, a greater proportion of CAS patients suffering from a perioperative MI died prematurely [27], making this yet another example of ‘spin over substance’.

To these methodological problems I would also add three others. First, is the impact of stopping an RCT too early. Stopped trials tend to happen on a random ‘high’ or ‘low’ and they are not always abandoned with statistically robust supporting evidence. Second is the fact that a significant proportion of RCTs are completed but never written up. This really is a scandal. In a recent review, 81 out of 395 RCTs listed on ClinicalTrials.gov were discontinued early, usually because of problems with recruitment. Of the 79 % which went on to completion, there was a publication rate of only 66 % at a median of 5 years after study completion. Industry sponsored RCTs were associated with lower odds of publication. This means that one in three completed RCTs went unpublished, thereby rendering it impossible for them to be included in subsequent meta-analyses [28]. This will, of course, limit the overall external validity of the trials. Third is the failure to accept that sometimes RCTs pass their ‘sell by date’ and become obsolete. Unfortunately, because many guideline bodies prioritize RCT data (to the exclusion of almost everything else), this means that they continue to be (inappropriately) upheld as examples of level 1, grade A evidence.

## What is the solution?

There is a very formalized and regulated structure for delivering new therapeutic drugs into clinical practice. This is in marked contrast to surgical interventions, which have largely been ‘unregulated and unstructured’ [29]. According to the IDEAL group, ’surgical innovation lacks major commercial funding sources for research, partly because device, implant and technology developers do not have the barriers to market entry as happens with drugs’ [29]. To address this (and also many of the other points raised in this review), the IDEAL group has proposed a series of recommendations for evaluating surgical innovations. The principle phases are highlighted in Table 1, which also indicates where roles for observational studies and RCTs might lie. As can be seen, the principles are quite similar to that advocated in the past but there are some key areas of change. For example, the IDEAL group recognizes that not all interventions need to be subject to RCT comparison. A contemporary example of this might include open versus endovascular repair for traumatic aortic transection. The IDEAL group also offers alternatives to RCT comparison in those situations where an RCT is considered inappropriate or unethical. Second, is recognition that the ‘learning curve’ must be considered more carefully during the planning phase of any RCT. Third, is a greater role for observational studies and well-designed registries to audit practice in the ‘real world’ to ensure that outcomes achieved in RCTs are being replicated in the community at large.

**Table 1:** The ‘IDEAL’ recommendations for evaluating new interventions^a^

**Stage 1**	New idea by a surgeon/interventionist	Published as a ‘first in man’ case report
**Stage 2a development phase**	Early report is positive and attracts early adopters. Focus is on technical development of the procedure	Prospective observational studies (OS)
**Stage 2b exploration phase**	Investigation of indications, potential benefits and harms. During this phase, the early adopters refine their skills and move further up the learning curve	Prospective research databases (RDs) and OS to inform development of any RCT. Any decision on RCTs **must** recognize the impact of any learning curve
**Stage 3 assessment phase**	Key question posed: “is this technique better than the existing one”. If the opportunity for robust evaluation is not seized upon, widespread adoption may happen without evidence	RCTs should remain the default option. RCT may not be necessary if the advance *CANNOT* be explained by chance or bias. Alternatives to RCT include: parallel group non-RCTs, controlled interrupted time series studies ,step wedge designs, tracker trials and expertise-based RCTs
**Stage 4 long-term study phase**	The procedure has become widely adopted. Essential to ensure that outcomes are audited and maintained within accepted standards. What is the level of external validity?	OS and registries with risk adjustment for patient comorbidities

^a^Adapted from McCulloch et al. [29].

## Future developments

### Prospective, randomized, controlled clinical registry trials

This is a new concept, pioneered by Sweden and other Scandinavian countries, whereby many of the practical and economic problems associated with RCTs can be alleviated by incorporating them within established clinical registries [30]. The recently published Thrombus Aspiration in ST Elevation MI in Scandinavia Trial (TASTE) is an excellent example of a new way of evaluating treatment strategies. Fundamental to this concept is the presence of a high-quality, clinical registry, in this case the Swedish Coronary Angiography and Angioplasty Registry (SCAAR), which is based on social security numbers and which has 100 % coverage of patients who remain in the country. It is funded by national health authorities (i.e. independent of industry) and offers an on-line platform for randomization, completion of case report forms and entering follow-up data (including those patients who declined to be randomized). In effect, the TASTE registry based RCT (i) combined the benefits of randomized treatment allocation as well as the best features of a large scale population based registry, (ii) it enabled broad inclusion criteria to be used, such as chest pain, < 24 h of onset, electrocardiogram (ECG) evidence of ST elevation MI and requirement for primary coronary intervention (PCI), which meant that the findings were more likely to represent ‘real world practice’, (iii) it greatly reduced trial expenditure and administration and (iv) enabled follow-up within an already existing registry structure.

The TASTE registry ultimately randomized 60 % of the approximately 12,000 patients presenting with an ST elevation MI undergoing PCI and showed that intracoronary thrombus aspiration before PCI did not reduce 30-day death/stroke/recurrent MI or stent thrombosis [30]. This is a model that could be adopted for use in a number of vascular and endovascular strategies in the future.

### The importance of post-RCT surveillance in the community

A large amount of time, effort and money is required to complete a RCT, yet there is little evidence that clinical governance ensures that ‘real world’ practices are safe and justifiable. An emerging example of this is the performance of CAS after the American Heart Association (AHA) liberalized CAS indications in 2011 to include ‘average risk for CEA’ symptomatic and asymptomatic patients [31]. The AHA recommended that CEA (CAS) be performed within centers with a 30-day death/stroke rate ≤ 6 % for symptomatic patients and ≤ 3 % for asymptomatic patients [31]; however, evidence from a recent systematic review of outcome data from contemporary, large-volume administrative datasets reporting outcomes following both CEA and CAS (predominantly from the USA), suggest that while CEA was performed with a procedural risk > 3 % in only 1 out of 15 registries in asymptomatic patients, 9 out of 15 registries (60 %) reported death/stroke rates in excess of 3 % after CAS [32]. More worrying, while all 18 registries reported that CEA was performed with a mean death/stroke rate < 6 % in ‘average risk for CEA’ symptomatic patients, 13 out of 18 registries (72 %) reported death/stroke rates > 6 %, while 5 out of 18 (28 %) reported death/stroke rates > 10 % [32]. Not one of these registries commented that any of these risks were excessive or how poor performance should be addressed [33].

Post-RCT surveillance of ‘real world’ outcomes is now a very important requirement of the IDEAL recommendations (Table 1) and examples of poor performance cannot be ignored any more. This is an ideal example of where observational studies and registries can rapidly inform hospital providers and health administrators as to whether their patients (as a whole) are receiving optimal care. Otherwise, there was little point in performing the RCT in the first place.

## Conclusion

It would be naïve to believe that the era of EBM is heading towards a decline; however, it is also naïve for advocates of using randomized trials to determine practice to ignore increasing problems relating to the development and performance of randomized trials in the modern era. Guideline bodies must revise their methodology to recognize the importance of well-performed observational studies so that these too can be incorporated within contemporary guidelines.

## References

[1] Winslow CM, Solomon DH, Chassin MR, Kosecoff J, Merrick NJ, Brook RH (1988). The appropriateness of carotid endarterectomy. N Engl J Med.

[2] Collaborators ECST (1998). Randomised trial of carotid endarterectomy for recently symptomatic carotid stenosis: final results of the European carotid Surgery Trial. Lancet.

[3] Barnett HJM, Taylor DW, Eliasziw M, Fox AJ, Ferguson GG, Haynes RB (1998). Benefit of carotid endarterectomy in patients with symptomatic moderate or severe stenosis. N Engl J Med.

[4] Belsey J What is evidence based medicine? www.medicine.ox.ac.uk/bandolier/painres/download/whatis/ebm.pdf. Accessed 26. June 2015

[5] Hannan EL (2008). Randomized clinical trials and observational studies: guidelines for assessing respective strengths and limitations. JACC Cardiovasc Interv.

[6] Booth CM, Tannock IF (2014). Randomised controlled trials and population-based observational research: partners in the evolution of medical evidence. Br J Cancer.

[7] Naylor AR, Sillesen H, Schroeder TV (2014). Clinical and imaging features associated with an increased risk of late stroke in patients with asymptomatic carotid disease. Eur J Vasc Endovasc Surg.

[8] Naylor AR, Sillesen H, Schroeder TV (2015). Clinical and imaging features associated with an increased risk of early and late stroke in patients with symptomatic carotid disease. Eur J Vasc Endovasc Surg.

[9] Anglemyer A, Horvath HT, Bero L (2014). Healthcare outcomes assessed with observational study designs compared with those assessed in randomized trials. Cochrane Database Syst Rev.

[10] Rothwell PM (2005). External validity of randomised controlled trials: “To whom do the results of this trial apply?”. Lancet.

[11] Chomowitz MI, Lynn MJ, Derdeyn CP, Turan TN, Fiorella D, Lane BF (2011). Stenting versus aggressive medical therapy for intracranial arterial stenosis. N Engl J Med.

[12] GALA Trial Collaborative Group (2008). General anaesthesia versus local anaesthesia for carotid surgery (GALA): a multicentre, randomised controlled trial. Lancet.

[13] Rerkasem K, Bond R, Rothwell PM (2004) Local versus general anaesthesia for carotid endarterectomy. Cochrane Database Syst Rev (2):CD000126. doi:10.1002/14651858 10.1002/14651858.CD000126.pub2 15106144

[14] Vaniyapong T, Chongruksut W, Rerkasem K (2013) Local versus general anaesthesia for carotid endarterectomy. Cochrane Database Syst Rev (12):CD000126. doi:10.1002/14651858.CD000126.pub4 10.1002/14651858.CD000126.pub4 24353155

[15] Barnett HJM, Barnes RW, Clagett GP, Ferguson GG, Robertson JT, Walker PM (1992). Symptomatic carotid artery stenosis: a solvable problem. The North American Symptomatic carotid Endarterectomy trial. Stroke.

[16] Veith FJ (2013). How can good randomized controlled trials in leading journals be so misinterpreted?. J Vasc Surg.

[17] Naylor AR, Ricco J-B (2013). The story of anybody, somebody, nobody and everybody. Eur J Vasc Endovasc Surg.

[18] Mas J-L, Chatellier G, Beyssen B, Branchereau A, Moulin T, Becquemin J-P (2006). Endarterectomy versus stenting in patients with severe symptomatic stenosis. N Engl J Med.

[19] International Carotid Stenting Study Investigators (2010). Carotid artery stenting compared with endarterectomy in patients with symptomatic carotid stenosis (International Carotid Stenting Study): an interim analysis of a randomised controlled trial. Lancet.

[20] Powell JT, Hinchliffe RJ, Thompson MM, Sweeting MJ, Ashleigh R, Bell R, on behalf of the IMPROVE Trial Investigators et al (2014) An endovascular strategy for suspected ruptured abdominal aortic aneurysm brings earlier home discharge but not early survival or cost benefits. Eur J Vasc Endovasc Surg 47:333–334 10.1016/j.ejvs.2013.12.017 24485843

[21] Reimerink JJ, Hoornweg LL, Vahl AC, Wisselink W, van den Broek TA, Legemate DA, for the Amsterdam Acute Aneurysm Trial Collaborators (2013). Endovascular repair versus open repair of ruptured abdominal aortic. Ann Surg.

[22] Desgranges P, Kobeiter H, Katsahians S, Bouffi M, Gouny P, Favre J-P (2015). ECAR (Endovasculaire ou Chirurgie dans les Anévrysmes aorto-iliaques Rompus): a French Randomized Controlled Trial of Endovascular Versus Open Surgical Repair of Ruptured Aorto-iliac Aneurysms. Eur J Vasc Endovasc Surg.

[23] Sweeting MJ, Ulug P, Powell JT, Balm R, for the Ruptured Aneurysm Trialists (2015). Ruptured aneurysm trials: the importance of longer-term follow-up and meta-analysis for 1 year mortality. Eur J Vasc Endovasc Surg.

[24] Yadav JS, Wholey MH, Kuntz RE, Fayad P, Katzen B, Mishkel MR, for the SAPPHIRE Investigators et al (2004) Protected carotid artery stenting versus endarterectomy in high-risk patients. N Engl J Med 351:1493–14501 10.1056/NEJMoa040127 15470212

[25] Naylor AR SAPPHIRE (2006). Precious gem or fools gold?. Vascular Dis Manag.

[26] Blackshear JL, Cutlip DE, Roubin GS, Hill MD, Leimgruber PP, Begg RJ (2011). Myocardial infarction after carotid stenting and endarterectomy results from the carotid revascularization endarterectomy versus stenting trial. Circulation.

[27] Naylor AR (2012). Hearts and minds. Eur J Vasc Endovasc Surg.

[28] Chapman SJ, Shelton B, Mahmood H, Fitzgerald JE, Harrison EM, Bhangu A (2014). Discontinuation and non-publication of surgical randomised controlled trials: observational study. BMJ.

[29] McCulloch P, Altman DG, Campbell WB, Flum DG, Glasziou P, Marshall JC, for the Balliol Collaboration et al (2009) No surgical innovation without evaluation: the IDEAL recommendations. Lancet 374:1105–1112 10.1016/S0140-6736(09)61116-8 19782876

[30] Frobert O, Lagerqvist B, Olivecrona GK, Omerovic E, Gudnason T, Maeng M (2013). Thrombus aspiration during ST-segment elevation myocardial infarction. N Engl J Med.

[31] Furie KL, Kasner SE, Adams RJ, Albers GW, Bush RL, Fagan SC (2011). Guidelines for prevention of stroke in patients with stroke transient ischemic attack: a guideline for healthcare professionals from the AHA/ASA. Stroke.

[32] Paraskevas K, Kalmykov E, Naylor AR (2015) Stroke/death rates following carotid artery stenting and carotid endarterectomy in contemporary administrative dataset registries: a systematic review. Eur J Vasc Endovasc Surg. doi:10.1016/j.ejvs.2015.07.032 10.1016/j.ejvs.2015.07.032 26346006

[33] Naylor AR (2015). The brighter the light the darker the shadow. Stroke.

